# Applying phylogenomics to understand the emergence of Shiga-toxin-producing *Escherichia coli* O157:H7 strains causing severe human disease in the UK

**DOI:** 10.1099/mgen.0.000029

**Published:** 2015-09-14

**Authors:** Timothy J. Dallman, Philip M. Ashton, Lisa Byrne, Neil T. Perry, Liljana Petrovska, Richard Ellis, Lesley Allison, Mary Hanson, Anne Holmes, George J. Gunn, Margo E. Chase-Topping, Mark E. J. Woolhouse, Kathie A. Grant, David L. Gally, John Wain, Claire Jenkins

**Affiliations:** ^1^​ Public Health England, 61 Colindale Avenue, London NW9 5EQ, UK; ^2^​ Animal Laboratories and Plant Health Agency, Woodham Lane, Surrey KT15 3NB, UK; ^3^​ Scottish E. coli O157/VTEC Reference Laboratory, Department of Laboratory Medicine, Royal Infirmary of Edinburgh, 51 Little France Crescent, Edinburgh EH16 4SA, UK; ^4^​ Future Farming Systems, R&D Division, SRUC, Drummondhill, Stratherrick Rd., Inverness IV2 4JZ, Scotland, UK; ^5^​ Centre for Immunity, Infection and Evolution, Kings Buildings, University of Edinburgh, Edinburgh EH9 3FL, UK; ^6^​ Division of Infection and Immunity, The Roslin Institute and Royal (Dick) School of Veterinary Studies, University of Edinburgh, Roslin EH25 9RG, UK; ^7^​ University of East Anglia, Norwich NR4 7TJ, UK

**Keywords:** emerging infections, *Escherichia coli*, genomics, One Health, public health microbiology, Shiga toxin

## Abstract

Shiga-toxin-producing *Escherichia coli* (STEC) O157:H7 is a recently emerged zoonotic pathogen with considerable morbidity. Since the emergence of this serotype in the 1980s, research has focussed on unravelling the evolutionary events from the *E. coli* O55:H7 ancestor to the contemporaneous globally dispersed strains observed today. In this study, the genomes of over 1000 isolates from both human clinical cases and cattle, spanning the history of STEC O157:H7 in the UK, were sequenced. Phylogenetic analysis revealed the ancestry, key acquisition events and global context of the strains. Dated phylogenies estimated the time to evolution of the most recent common ancestor of the current circulating global clone to be 175 years ago. This event was followed by rapid diversification. We show the acquisition of specific virulence determinates has occurred relatively recently and coincides with its recent detection in the human population. We used clinical outcome data from 493 cases of STEC O157:H7 to assess the relative risk of severe disease including haemolytic uraemic syndrome from each of the defined clades in the population and show the dramatic effect Shiga toxin repertoire has on virulence. We describe two strain replacement events that have occurred in the cattle population in the UK over the last 30 years, one resulting in a highly virulent strain that has accounted for the majority of clinical cases in the UK over the last decade. There is a need to understand the selection pressures maintaining Shiga-toxin-encoding bacteriophages in the ruminant reservoir and the study affirms the requirement for close surveillance of this pathogen in both ruminant and human populations.

## Data summary

1. FASTQ sequences were deposited in the NCBI Short Read Archive under the BioProject PRJNA248042 (http://www.ncbi.nlm.nih.gov/bioproject/?term = PRJNA248042)
2. Supplementary Table 5 is available at the following git repository: https://github.com/timdallman/phylogenomics_stec.git

## Impact statement

In this article, we analyse over 1000 Shiga-toxin-producing *Escherichia coli* (STEC) O157:H7 genomes from animal and clinical isolates collected over the past three decades and present for the first time a comprehensive population structure of STEC O157:H7. Using phylogenetic methods we have examined the origin and dispersal of this zoonotic pathogen and show how historical worldwide dissemination followed by regional expansion in native cattle populations gave rise to the extant diversity seen today. By comparing clinical outcome data of nearly 500 human cases we comprehensively assess the association between phylogenetic grouping, acquisition and loss of specific subtypes of Shiga toxin, and severe disease. With this analysis, we show specific circulating strains carry > fivefold increased risk of severe disease than the ancestral STEC O157:H7 genotype. Finally, we show that recent strain replacement has occurred in Great Britain shaping the diversity of STEC O157:H7 observed today and introducing a high virulence clone into the British cattle population.

## Introduction

Shiga-toxin-producing *Escherichia coli* (STEC) O157:H7 is a globally dispersed pathogen that, whilst generally asymptomatic in its ruminant host, can cause severe outbreaks of gastroenteritis, haemorrhagic colitis and haemolytic uraemic syndrome (HUS) in humans [Akashi *et al.*, 1994; Centers for Disease Control and Prevention (CDC), 2006; Ihekweazu *et al.*, 2012]. Contemporary STEC O157:H7 represent a monomorphic clone (Whittam *et al.*, 1988) characterized by particular phenotypic properties including the inability to ferment sorbitol and produce β-glucuronidase. Over the course of its evolution, STEC O157:H7 has acquired several virulence determinants including two types of Shiga toxins (Stx1 and Stx2) encoded on lambdoid bacteriophages (Scotland *et al.*, 1985), a myriad of effector proteins (Lai *et al.*, 2013; Tobe *et al.*, 2006) and a virulence plasmid containing genes for a type II secretion system and a haemolysin (Schmidt *et al.*, 1994). It is postulated that the current clone arose with the transfer of the O157 *rfb* and *gnd* genes that specify the structure of lipopolysaccharide side-chains that comprise the somatic (O) antigens into a *stx2* containing *E. coli* O55:H7 strain that had an enhanced capacity for host colonization mediated by the locus of enterocyte effacement (LEE) pathogenicity island (Wick *et al.*, 2005). A step-wise sequence of events involving the loss of the ability to utilize sorbitol, lysogenization by an *stx1*-containing phage and inactivation of the gene encoding the β-glucuronidase *uidA* is hypothesized to have given rise to the currently circulating clone (Feng *et al.*, 1998), with distinct subpopulations formed by less common non-motile O157:H strains and strains that retained the ability to express β-glucuronidase.

Despite high levels of relatedness of the non-sorbitol-fermenting, β-glucuronidase-negative STEC O157:H7 strains, it has long been realized that distinct lineages exist within the population. It is suggested that these arose from the result of geographical spread of an ancestral clone and subsequent regional expansion (Kim *et al.*, 2001; Yang *et al.*, 2004). Identified subpopulations have also been found to be unequally distributed in the cattle and human populations with lineage I being more prevalent among human clinical isolates and lineage II more associated with the animal host (Yang *et al.*, 2004). Subsequent studies revealed differences between the two lineages including Stx-encoding bacteriophage (Stxϕ) insertion sites (Besser *et al.*, 2007), *stx2* expression (Dowd & Williams, 2008) and stress resistance (Lee *et al.*, 2012), as well as lineage-specific polymorphisms (Bono *et al.*, 2007). Further characterization of genomic differences between these two lineages identified an intermediate genogroup termed lineage I/II (Zhang *et al.*, 2007). To investigate the propensity of different STEC O157:H7 strains to cause serious illness, further subtyping schemes have been developed which subdivided the population into nine clades based on single nucleotide polymorphisms (SNPs; Manning *et al.*, 2008; Riordan *et al.*, 2008) with clade 8 associated with two large outbreaks of HUS (Manning *et al.*, 2008). Subsequent *in vitro* studies showed varied adherence and virulence factor expression between different clades (Abu-Ali *et al.*, 2010) and whole genome studies elucidated further potential virulence determinants (Eppinger *et al.*, 2011a). The use of clade genotyping provided further evidence that the diversity within STEC O157:H7 is globally distributed (Mellor *et al.*, 2013; Yokoyama *et al.*, 2012).

Several groups have used the clade description of the STEC O157:H7 population to further speculate on the evolutionary path that has given rise to the current diversity (Kyle *et al.*, 2012; Leopold *et al.*, 2009; Yokoyama *et al.*, 2012). The current model suggests that β-glucuronidase-positive, non-sorbitol-fermenting STEC O157:H7 (clade 9) are ancestral to lineage II and the intermediate lineage I/II (which overlap with clades 8–5) which themselves are ancestral to lineage I (clades 5–1). The nature of the paraphyletic evolution of these lineages, however, remains unknown.

**Fig. 1.**
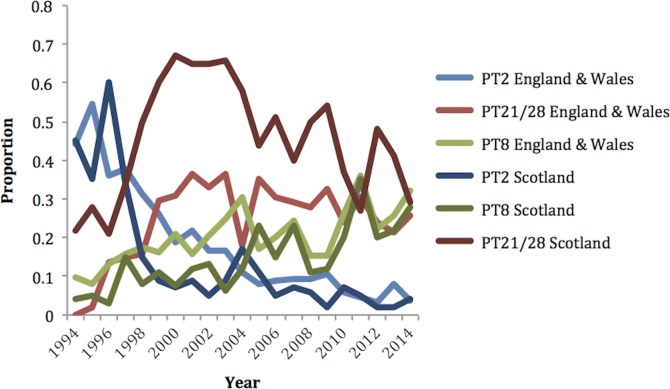
Proportion of cases of the predominant phage types in England and Wales, and Scotland over the last 20 years.

The UK has a comparatively high human infection rate with STEC O157 (Chase-Topping *et al.*, 2008) and this has remained relatively constant over the last decade. In the UK, STEC O157 strains are subtyped by determining sensitivity to a specific panel of 16 typing phages, a phage typing scheme developed in Canada and adopted by several European countries (Ahmed *et al.*, 1987; Khakhria *et al.*, 1990). Over the last decade in England, Scotland and Wales, phage type (PT) 21/28 strains have been most commonly associated with severe human infection, and more recent research has indicated that these strains are more likely to be associated with high excretion levels from cattle, known as supershedding (Chase-Topping *et al.*, 2008). Previously, the most common phage type in England, Scotland and Wales was PT2 until it decreased year after year from 1998 (see Fig. 1). The nature of this strain replacement and how PT21/28, PT2 and other common phage types, such as PT8 and PT32, are associated with each other and with the lineages defined above was not understood. In this study we present the population structure of STEC O157:H7 from a UK perspective using genome sequencing of over 1000 animal and clinical isolates collected over the past three decades. Using phylogenetic methods we have examined the origin and dispersal of this zoonotic pathogen and estimated approximate evolutionary timescales that have led to the emergence of an expanded virulent cluster that accounts for a significant proportion of the human STEC disease in the UK.

## Methods

### Strain selection

A total of 1075 strains of STEC O157 from clinical and animal isolates from England, Northern Ireland, Wales and Scotland collected from 1985 to 2014 were selected for sequencing. These represented 25 phage types. Ninety-five cattle strains were STEC O157:H7 isolates selected for sequencing from Scottish cattle strains collected as part of ‘The Wellcome Foundation International Partnership Research Award in Veterinary Epidemiology’ (IPRAVE) study on the basis of regional and genotypic diversity. Fifty-four sequences were downloaded from public repositories including the oldest sequenced STEC O157 (Sanjar *et al.*, 2014).

### Genome sequencing and sequence analysis

Genomic DNA was fragmented and tagged for multiplexing with Nextera XT DNA Sample Preparation kits (Illumina) and sequenced at the Animal Laboratories and Plant Health Agency using the Illumina GAII platform with 2 × 150 bp reads. Short reads were quality-trimmed (Bolger *et al.*, 2014) and mapped to the reference STEC O157 strain *Sakai* (GenBank accession BA000007) using BWA-SW (Li & Durbin, 2010). The sequence alignment map output from BWA was sorted and indexed to produce a binary alignment map (BAM) using Samtools (Li *et al.*, 2009). GATK2 (McKenna *et al.*, 2010) was used to create a variant call format (VCF) file from each of the BAMs, which were further parsed to extract only SNP positions which were of high quality (MQ ≥ 30, DP ≥ 10, GQ ≥ 30, variant ratio ≥ 0.9). Pseudosequences of polymorphic positions were used to reconstruct maximum-likelihood trees using RaxML (Stamatakis, 2014). Pairwise SNP distances between each pseudosequence were calculated. Spades version 2.5.1 (Bankevich *et al.*, 2012) was run using careful mode with kmer sizes 21, 33, 55 and 77 to produce *de novo* assemblies of the sequenced paired-end fastq files.

### SNP clustering

Hierarchical single linkage clustering was performed on the pairwise SNP difference between all strains at various distance thresholds (Δ250, Δ100, Δ50, Δ25, Δ10, Δ5, Δ0). The result of the clustering is a SNP address that can be used to describe the population structure based on clonal groups.

### Recombination

Recombination analysis was performed using bratnextgen (Marttinen *et al.*, 2012). Representatives from Δ50 SNP clusters were randomly selected and whole-genome alignments produced relative to the reference strain *Sakai*. From the proportion of shared ancestry generated by bratnextgen, the dataset was partitioned into 18 clusters. Recombination between and within these clusters was calculated over 20 iterations and the significance estimated over 100 replicates. Recombinant segments detected were deemed significant with a *P* value < 0.05.

### Timed phylogenies

Timed phylogenies were reconstructed using beast-mcmc v1.80 (Drummond *et al.*, 2012) and after first confirming a temporal signal using Path-O-Gen (Drummond *et al.*, 2012). Alternative clock models and population priors were computed and their suitability assessed based on Bayes factor (BF) tests. The highest supported model was a relaxed lognormal clock rate under a constant population size. All models were run with a chain length of one billion. A maximum clade credibility tree was reconstructed using TreeAnnotator v1.75.

### Shiga toxin subtyping

Shiga toxin subtyping was performed as described by Ashton *et al.* (2015).

### Stx-associated bacteriophage insertion (SBI)

The integration of Shiga-toxin-carrying prophage into the host genome has been characterized into six target genes: *wrbA* (Hayashi *et al.*, 2001), which encodes a NADH quinone oxidoreductase; *yehV* (Yokoyama *et al.*, 2000), a transcriptional regulator; *sbcB* (Ohnishi *et al.*, 2002), an exonuclease; *yecE*, a gene of unknown function; the tRNA gene *argW* (Eppinger *et al.*, 2011a); and Z2577, which encodes an oxidoreductase. Intact reference sequences of these genes were obtained and compared by blastn blast(Altschul *et al.*, 1990) against the STEC O157:H7 genome assemblies. Occupied SBI sites were defined as those strains that had disrupted blast alignments.

### Clade typing

Clade typing was performed as originally defined by Manning *et al.* (2008). The eight definitive polymorphic positions adopted by Yokoyama *et al.* (2012) were used to delineate the strains into the nine clade groupings.

### Locus-specific polymorphism assay – LSPA6

Based on the polymorphic genes defined by Yang *et al.* (2004), reference sequences of six genes were extracted from the *Sakai* reference genome. Sequence alignments were generated using blastn of these sequences against the STEC O157:H7 genome assemblies. The allelic designation ‘1’ was assigned to WT, ‘2’ assigned to the insertions/deletions defined by Yang *et al.* (2004) and ‘X’ to all other polymorphisms.

Each allele (*folD-sfmA*, *Z5935*, *yhcG*, *rbsB*, *rtcB* and *arp-iclR*) was assigned a number as described previously (Yang *et al.*, 2004). Isolates showing the LSPA6 genotype 1-1-1-1-1-1 were classified as LSPA6 lineage I (LSPA6 LI), while those with LSPA6 genotype 2-1-1-1-1-1 were classified as LSPA6 lineage I/II (LSPA6 LI/II). Unique alleles (aberrant amplicon size) were assigned new numbers. All deviations from the genotypes 1-1-1-1-1-1 and 2-1-1-1-1-1 were classified as LSPA6 lineage II (LSPA6 LII).

### Statistical analyses of clinical data amongst clinical cases reported in England

The National Enhanced Surveillance System for STEC (NESSS) in England was implemented on 1 January 2009, and has been described in detail elsewhere (Byrne *et al.* 2015). In brief, it collates standardized demographic, clinical and exposure data on all cases of STEC reported in England through collection of a standard enhanced surveillance questionnaire (ESQ). For this study, clinical data on clinical cases for whom strains were sequenced were extracted from NESSS. These data included whether the case reported symptoms of non-bloody diarrhoea, bloody diarrhoea, vomiting, nausea, abdominal pain, fever or whether they were an asymptomatic carrier detected through screening high-risk contacts of symptomatic cases. Data on whether cases were hospitalized, developed typical HUS or died were also extracted. The age and gender of cases were also extracted. Where clinical symptoms were blank on the ESQ and cases were not recorded as being asymptomatic, these were coded as negative responses. Cases were categorized into children (aged 16 and under) or adults, based on a priori knowledge that children are most at risk of both STEC infection and progression to HUS (Byrne *et al.*, 2015). While adults aged over 60 are at increased risk of STEC infection and development of HUS, they were under-represented in these data and were not analysed as a separate group. The outcome of interest was disease severity. Cases were coded as having severe disease if any of the following criteria were reported: bloody diarrhoea, hospitalization, HUS or death. Asymptomatic cases and cases with non-bloody diarrhoea were classed as mild.

Genomic variables for analyses included Stx subtype and sublineage. Sublineages were described with respect to Stx subtypes. Cases were described with respect to clinically mild or severe disease and HUS separately by sublineage. Disease severity was compared amongst gender and age of cases, and sublineage, and Fisher's exact tests were used to compare proportions. Logistic regression analysis was used to investigate phylogenetic groups associated with more severe disease outcomes. Due to the correlation between Stx subtypes and lineage, sublineage was chosen as an explanatory variable for analyses. To assess whether there was a difference in disease severity within sublineages, they were further subdivided by Stx subtype for analysis. Odds ratios for cases reporting severe disease compared with those reporting mild disease were calculated for each variable. Lineage IIa was chosen as the baseline for lineages as it was found to be the ancestral O157 lineage.

## Results

### Phylogeny of STEC O157 in the UK

A maximum-likelihood (ML) phylogeny (Fig. S1 available in the online Supplementary Material) revealed the population structure of the STEC O157 isolates sequenced in this study. The STEC O157:H7 population has previously been delineated into three lineages, I, I/II and II (Feng *et al.*, 1998; Zhang *et al.*, 2007), and the phylogeny presented here also splits the strains into three groups via deep branches, with reference strains of known lineage (Eppinger *et al.*, 2011b) conforming to the expected pattern.

The ML phylogeny was compared with two other previously used methods to describe the STEC O157 population, namely LSPA6 type (Yang *et al.*, 2004) (Fig. S1a) and the Manning clade typing scheme (Manning *et al.*, 2008) (Fig. S1b). LSPA6 typing was not congruent with the phylogeny and the lineages defined by LSPA type do not reflect the phylogenetic clustering generated on polymorphisms across the whole genome. By LSPA6, the only strains that typed as lineage I (LSPA6 1-1-1-1-1-1) were a clade containing the lineage I strain the assay was designed upon, EDL933. Other strains that cluster within this deep branch (and therefore should be of the same lineage) typed as lineage I/II (LSPA6 2-1-1-1-1-1) or had a novel polymorphism. Similarly across the rest of the ML phylogeny, the predominant LSPA6 was 2-1-1-1-1-1 or a novel polymorphism. Based on this population, LSPA6 typing did not resolve the lineages correctly and, therefore, we defined the lineages I, I/II and II on the basis of the deep phylogenetic branches and the placement of reference strains of known lineage.

Fig. S1b shows the phylogeny coloured by clades as described by Manning *et al.* (2008). The clade groupings were broadly congruent, with the phylogeny clade 7 (purple), clade 8 (pink) and clade 4/5 (blue) predominating and clade 9 (bright green), comprising strains that were β-glucuronidase-positive, as an outgroup. It was clear, however, that clade typing does not resolve many phylogenetic splits. In terms of clade typing, lineage II corresponded to clade 7, lineage I/II corresponded to clade 8 and lineage I corresponded to clades 6 through 1 as suggested previously (Eppinger *et al.*, 2011a).

Single linkage clustering based on pairwise genetic distance is an effective method of defining phylogenetic groups as it is inclusive of clonal expansion events. Using a SNP distance threshold of Δ250 we clustered the 1224 strains in this study into 54 groups. Of these, 52/54 clusters were distributed within the three lineages and there were two outlier clusters, one contained the β-glucuronidase-positive strains and another contained three isolates associated with travel to Turkey. Fig. S2 shows the number and size of the 52 clusters within the three lineages. Lineage II contained the most diversity with 32 clusters whilst lineage I and lineage I/II contained 17 and 3 clusters, respectively. All three lineages were associated with uneven sampling of diversity with single high-density clusters comprising 77 % of lineage I isolates, 73 % of lineage I/II isolates and 47 % of lineage II isolates. Isolates contained within the high-density clusters in lineage I, I/II and II represented the common phage types associated with human infection in the UK: PT21/28, PT2 and PT8, respectively. Isolates in clusters with five or less representatives were more likely to be non-UK strains associated with foreign travel or imported food. Ninety-five isolates were from cattle faecal pats collected as part of a large survey in Scotland (Pearce *et al.*, 2009). These cattle isolates were present in only 8/54 clusters across the three lineages with 84 % found in the three high-density clusters identified above. This pattern of uneven diversity, coupled with the association of domestic cattle with high-density clones, supports the model of global dispersion and regional expansion of STEC O157:H7.

### Recombination

**Fig. 2.**
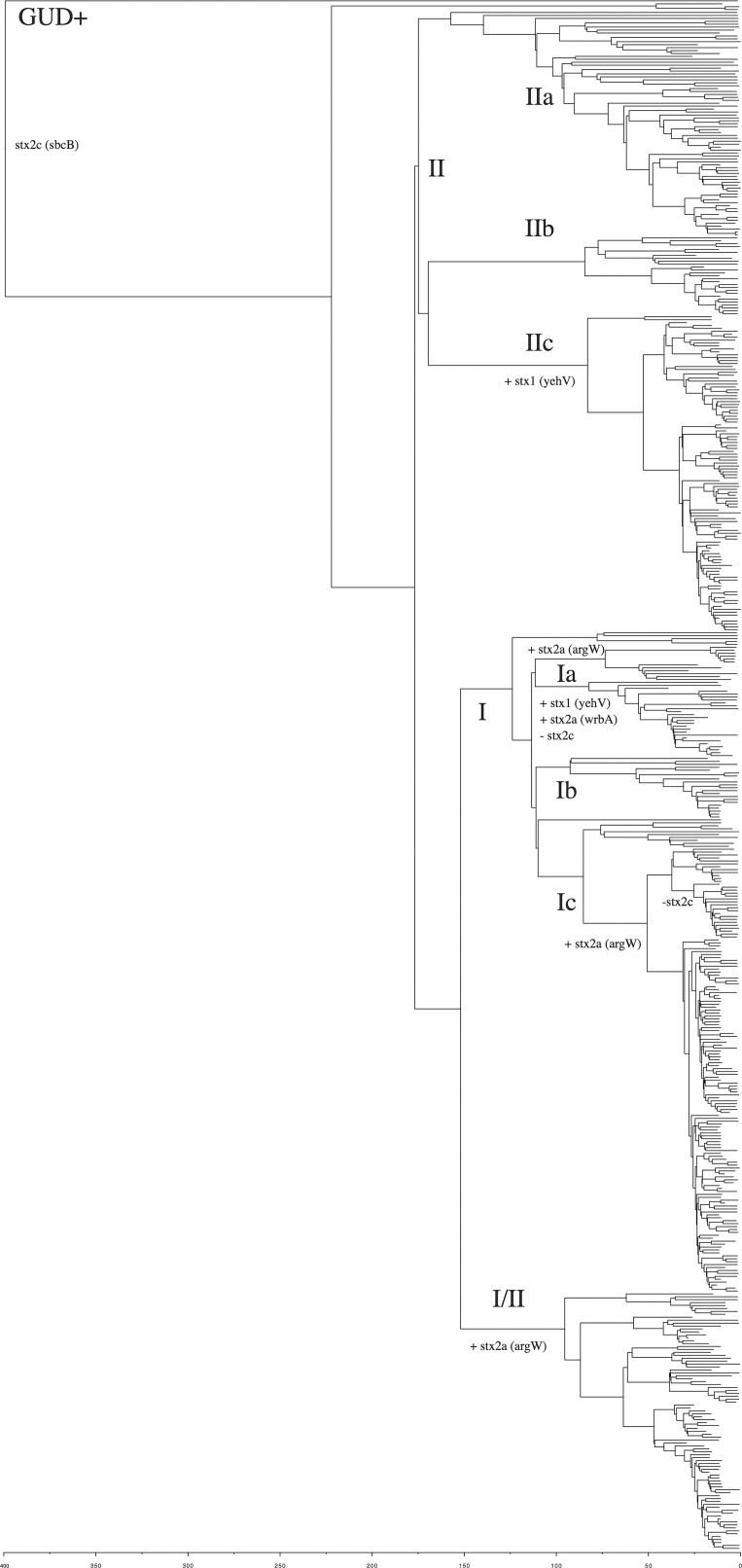
Maximum clade credibility tree of 530 Δ25 SNP representatives. The tree is highlighted by lineage and the loss and gain of Stxϕ with the associated SBI in parentheses. The GUD+ lineage represents the strains that retained the ability to express β-glucuronidase. Scale is in years.

Signals of recombination in the sample population were analysed with bratnextgen using 270 Δ50 SNP threshold cluster representatives. There were 631 016 recombinant positions found across the 5 498 450 bp alignment and 90 % had their origin in the 18 *Sakai* prophages (SP) or 6 *Sakai* prophage like elements (SPLE) suggesting that almost all genetic transfer (at least historical) was phage-mediated. The median recombinant size was 575 bp whilst the largest was 41 212 nt representing an intra-lineage II recombination of SP1. Recombination events were seen at least twice as frequently within lineages (Table S1) than between lineages, with no statistical difference association between the lineage and its likelihood to be a donor or recipient. Within lineage II, the ancestral lineage (see Fig. 2) lineage IIa appeared to be the donor of most recombination events with lineage IIc only receiving foreign DNA. Lineage I had the highest intra-lineage recombination rate, and this could have contributed to the heterogeneous *stx* complement as described in more detail below.

### Evolutionary timescale and Stx prophage insertion in STEC O157

A timed phylogeny was reconstructed using beast (Fig. 2). The mutation rate of STEC O157:H7 was calculated to be approximately 2.6 mutations genome^− 1^ year^− 1^ [95 % highest posterior density (HPD), 2.4–2.8], which is in-line with previous estimates for *E. coli* (von Mentzer *et al.*, 2014) and closely related *Shigella* species (Holt *et al.*, 2012). We predict the split of the contemporary β-glucuronidase-negative, sorbitol-negative clone from the β-glucuronidase-positive ancestor to be approximately 400 years ago (95 % HPD, 520 years–301 years). The time to common ancestor of the current circulating diversity (e.g. lineage I, I/II and II) is approximately 175 years (95 % HPD, 198 years–160 years), significantly more recent than previous estimates of 400 years (Yang *et al.*, 2004) and 2500 years (Leopold *et al.*, 2009). Lineage II is the ancestral lineage, which contains at least three sublineages that diverged early in the evolutionary process. The most recent common ancestor to lineage I and lineage I/II existed approximately 150 years ago (95 % HPD, 175 years–130 years).

The model of Shiga toxin acquisition proposed by Wick *et al.* (2005) and Feng *et al.* (1998) suggested the acquisition of a lambdoid phage containing *stx2* followed by the later acquisition of an *stx1*-containing-phage (Stx1ϕ). The timed phylogeny supported this hypothesis (Fig. 2) as the β-glucuronidase-positive ancestor and the majority (70 %) of strains within lineage IIa and IIb contained only *stx2c*. Sublineage lineage IIc (PT8) (Fig. 2) was subsequently lysogenized by an Stx1ϕ and had the same disrupted Shiga toxin insertion targets, *yehV* and *sbcA*, supporting the hypothesis that a truncated prophage was replaced with a Stx1ϕ in *yehV* (Shaikh & Tarr, 2003).

The majority of strains in Lineage IIb (PT4/PT1) (Fig. 2) carried *stx2c* only but had an occupied *argW* SBI site. There was some further observed heterogeneity in the ancestral lineage IIa with small numbers of dispersed strains containing Stx1ϕ, Stx2ϕa or being negative for any Shiga toxin alleles as well as having non-Stx disrupted SBI sites (Table S2).

The common ancestor of lineage I/II (Fig. 2) was approximately 95 years old, marking the divergence of the strain that caused the 2006 Taco Bell outbreak in North America (Sodha *et al.*, 2011) and the PT2 strains associated with the first outbreak of HUS in the UK in 1983 (Taylor *et al.*, 1986). The majority (65 %) of strains in lineage I/II were positive for both *stx2c* and *stx2a* with occupied SBIs at *yehV*, *sbcA* and *argW*. One sub-group of strains belonging to PT2 have subsequently lost Stx2cϕ and had an intact *sbcA* (Table S3).

Lineage I was by far the most heterogeneous in terms of Stx complement (Table S4) and arose from a *stx2c*-only ancestor approximately 125 years ago (Fig. 2). The majority (87 %) of strains in Lineage Ib (PT32) retained the ancestral *stx2c* only genotype of lineage II and had an additional *yecE* SBI occupied. This lineage had an overrepresentation of strains from Scottish cattle and very few clinical strains. The majority (64 %) of strains in lineage Ia contained Stx2aϕ and Stx1ϕ with disrupted *yehV* and *wrbA* including the first fully sequenced STEC O157:H7 genomes [*Sakai* (Hayashi *et al.*, 2001) and Edl-933 (Latif *et al.*, 2014)] and the genome sequence of *E. coli* O157:H7 strain 2886-75, which was isolated in 1975 making it the oldest STEC O157:H7 strain for which a genome sequence is available (Sanjar *et al.*, 2014). Lineage Ia also contained strains that typed as clade 6 by the Manning scheme and carry the *stx2c* and *stx2a* genes with disrupted *yehV* and *sbcA*, which suggests either Stx2aϕ inserted into *yehV* or a novel insertion site.

A final sublineage of lineage I (lineage Ic) contained 40 % of the strains in this study and its common ancestor was approximately 50 years old and had since diverged into three clades. These included the ancestral *stx2c*-only genotype with occupied *yehV* and *sbcA* SBIs, a *stx2a*-only genotype with occupied *yecE* and *yehV* insertion sites, and a *stx2a* and *stx2c* genotype with occupied SBIs *yehV*, *sbcA* and *argW*. This final genotype was predominated by phage type 21/28. Within the PT 21/28 clade, a subclade had subsequently lost the *stx2c* toxin although *yehV*, *sbcA* and *argW* remain occupied.

All 1129 genomes analysed in this study are summarized in terms of lineage, SNP cluster, SBI, *stx* type, Manning Clade and LSPA-6 type in Table S5.

### Recent emergence of predominant UK lineages

The phage types PT8 and PT21/28 accounted for approximately 60 % of clinical isolates identified in the UK in 2014. Phage typing of STEC O157:H7 in the UK suggests strain replacement has occurred since the beginning of the 21st century with a decline in PT2 corresponding with a rise in PT21/28. PT2 was restricted to lineage I/II whereas PT21/28 was restricted to lineage I indicating strain replacement of one genotype by another distinct genotype, rather than phage type switching within a single genotype.

**Fig. 3.**
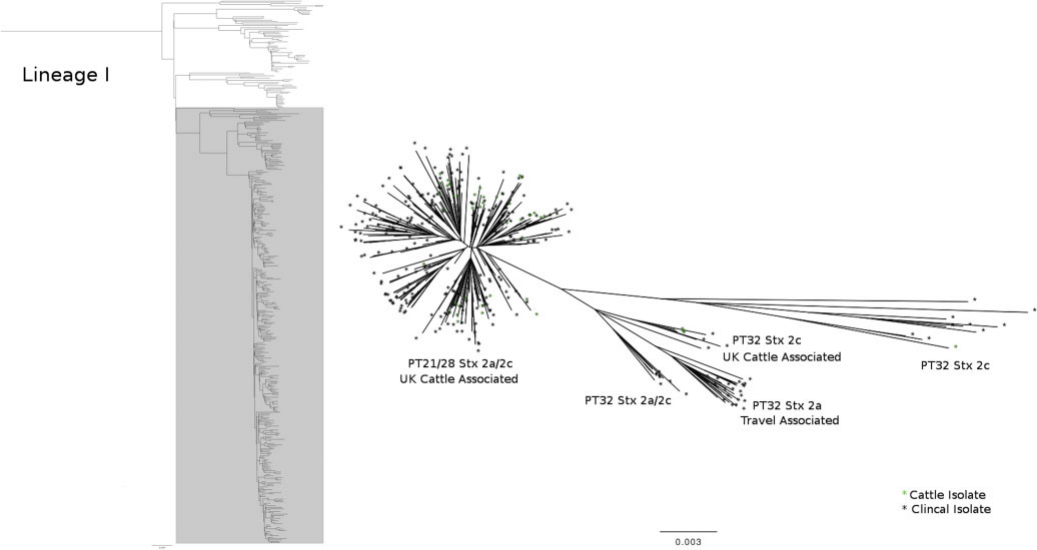
Left, maximum-likelihood phylogeny of 400 lineage I Δ5 SNP representatives with lineage Ic highlighted in grey. Right, maximum-likelihood phylogeny of lineage Ic showing the radial expansion of PT21/28 from the PT32 ancestor with isolates annotated by cattle or clinical origin. Scale represents substitutions per site.

PT 21/28 typically accounts for >30 % of clinical isolates seen in the England, Wales and Scotland each year and is the phage type most commonly associated with outbreaks of HUS (Underwood *et al.*, 2013). As stated above, divergence from the most recent common ancestor occurred 50 years ago subsequently created 3 clades: the ancestral PT32 *stx2c*-only genotype, a *stx2a*-only PT32 genotype associated with travel to Ireland and mainland Europe and finally the PT21/28 clade as a single Δ50 SNP cluster. The PT21/28 clade contained a large number of British cattle (57 % of total cattle isolates) and clinical isolates but very few isolates associated with foreign travel ( < 1 %). The PT21/28 clade arose only 25 years ago and has since undergone a radial expansion resulting in a ‘comet’ like phylogeny (Fig. 3). The PT 21/28 clade itself was flanked by three PT32 *stx2a* and *stx2c* isolates, two from cattle and one clinical isolate from Scotland. It is clear that the direct ancestor of PT21/28 is a PT32 strain.

**Fig. 4.**
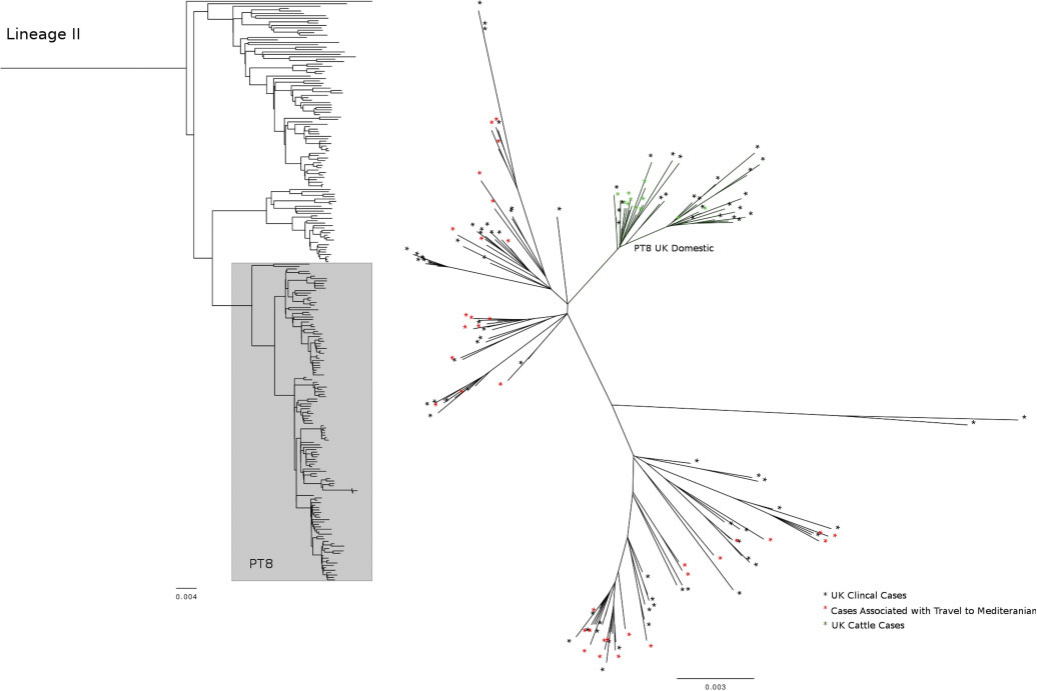
Left, maximum-likelihood phylogeny of 241 lineage II Δ5 SNP representatives with lineage IIc (PT8) highlighted in grey. Right, maximum-likelihood phylogeny of lineage IIc showing the distribution of Mediterranean-travel-associated cases and UK cattle cases. Scale represent substitutions per site.

PT8 was represented as a single Δ250 SNP clonal group (lineage IIc) and its most recent common ancestor can be dated to approximately 50 years ago. Across this clonal group, cases were associated with travel to southern Europe and northern Africa (22 %) suggesting this strain may be endemic in cattle in this region. Within this group there was a recently emerged (30 years to most recent common ancestor) subclade where several cases reported exposure to domestic cattle, cases reported no foreign travel, and there were several strains from UK cattle, suggestive of a domestic source of human infection (Fig. 4). This again highlights the possibility of imported strains of O157:H7 becoming endemic in local cattle populations.

### Disease severity of clinical cases in England by *stx* subtype and sublineage

A total of 493 strains from clinical cases in England had clinical data available in NESSS. Of those, 311 (63.1 %) had experienced bloody diarrhoea, 158 (32.0 %) had been hospitalized with their illness and 26 (5.3 %) were from cases known to have developed HUS. Thus, two thirds of cases in the dataset were categorized as having severe disease (as defined in methods); however, this varied by *stx* subtype and sublineage (Table 1). Cases classed as having mild disease accounted for 33.5 % of the dataset, and included 18 asymptomatic cases. Over half (55.4 %) of cases in the dataset were female and 55.2 % were children aged 16 and under. Severe disease was more frequently reported amongst females (70.3 % versus 29.7 %, *P* = 0.044) and children (71.9 % versus 28.1 %, *P* = 0.005).

**Table 1.** Sublineage and *stx* subtype of whole-genome-sequenced strains isolated from clinical cases of STEC O157 in England

Sublineage	Mild		Severe*		Total		HUS†	
	*n* (%)		*n* (%)		*n* (%)		*n* (%)	
II a	42	56.8	32	43.2	74	100	1	1.4
II b	18	81.8	4	18.2	22	100	0	0.0
II c	31	23.7	100	76.3	131	100	1	0.8
I a	3	17.7	14	82.3	17	100	0	0.0
I b	7	77.8	2	22.2	9	100	0	0.0
Ic (*stx2a*)	9	20.9	34	79.1	43	100	8	18.6
Ic (*stx2a*/*2c*)	35	30.2	81	69.8	116	100	10	8.6
Ic (*stx2c*)	1	25.0	3	75.0	4	100	0	0.0
I/II (*stx2a*)	7	18.4	31	81.6	38	100	2	5.3
I/II (*stx2a*/*2c*)	12	30.8	27	69.2	39	100	4	10.3
All strains	165	33.5	328	66.5	493	100	26	5.3

* Includes cases with bloody diarrhoea or cases who were hospitalized.

† The lineage IIa strain isolated from a patient with HUS possessed *stx2a/2c*; the lineage IIc strain possessed *stx1a/2a/2c*.

In univariable analysis, being a child and being female were significantly associated with severe disease (Table 2). All sublineages, except Ib and Ic carrying *stx2c*, were significantly associated with more severe disease as compared with sublineage IIa. In the final multivariable model when all variables were controlled for, being a child was a significant predictor of severe disease, but being female was no longer significant. Sublineage Ia had the greatest odds of severe disease, with a sixfold-increased odds as compared with IIa.

**Table 2.** Disease severity amongst clinical cases of STEC O157 in England, where strains had been whole-genome-sequenced, by age, gender and sublineage

Variable	Category	Odds ratio	*P* value	Lower 95 % CI	Upper 95 % CI
**Univariable analysis**					
Age	Child	1.73	0.005	1.18	2.51
	Adult	Baseline			
Sex	Female	1.49	0.037	1.02	2.17
	Male	Baseline			
Sublineage	IIa	Baseline			
	IIb	0.29	0.040	0.09	0.95
	IIc	4.23	0.000	2.30	7.80
	Ia	6.12	0.008	1.62	23.14
	I b	0.37	0.240	0.07	1.93
	Ic (*stx2a*)	4.96	< 0.001	2.08	11.80
	Ic (*stx2a*/*2c*)	2.92	0.001	1.59	5.34
	Ic (*stx2c*)	3.94	0.245	0.39	39.65
	I/II (*stx2a*)	5.81	< 0.001	2.27	14.88
	I/II (*stx2a*/*2c*)	2.95	0.010	1.30	6.71
**Multivariable analysis**					
Age	Child	1.56	0.042	1.01	2.39
	Adult	Baseline			
Sex	Female	1.15	0.489	0.76	1.75
	Male	Baseline			
Sublineage	IIa	Baseline			
	IIb	0.29	0.040	0.09	0.95
	IIc	3.65	< 0.001	1.95	6.83
	Ia	6.09	0.008	1.60	23.20
	Ib	0.35	0.209	0.67	1.81
	Ic (*stx2a*)	5.05	< 0.001	2.11	12.10
	Ic (*stx2a*/*2c*)	3.06	< 0.001	1.66	5.67
	Ic (*stx2c*)	3.48	0.293	0.34	35.62
	I/II (*stx2a*)	4.89	0.001	1.88	12.73
	I/II stx (*stx2a*/*2c*)	2.87	0.012	1.26	6.58

All but one of the HUS cases fell within sublineages Ic and I/II (Fig. 1) and all were infected with strains carrying *stx2a* either alone or with *stx2c* (Table 2). Lineages Ic and I/II were further divided into strains possessing *stx2a* only and those with *stx2a*/*2c*. Across all strains, there was no difference in disease severity between cases infected with strains carrying *stx2a* alone or with *2c* (53.5 % versus 46.5 %, *P* = 0.291). However, in both sublineages Ic and I/II, strains carrying *stx2a* only had higher odds of severe disease than those carrying *stx2a*/*2c* in the final model. While sublineage IIc had increased odds of severe disease, no cases developed HUS. Rather, this was due to increased reporting of bloody diarrhoea amongst cases infected with these strains compared with those in other sublineages (75.6 % versus 58.6 % in other sublineages, *P* = 0.005). Most strains (92 %) in this sublineage carried *stx1a*/*2c*. Overall, cases infected with strains carrying *stx1a* reported bloody diarrhoea more frequently than those without (77.5 % versus 61.8 %, *P* = 0.001) leading to the hypothesis that possession of *stx1a* in strains of sublineage IIc leads to higher rates of bloody diarrhoea.

## Discussion

Using phylogenetic analysis of variation at the whole genome level we have been able to reconstruct the phylogenetic history and global diversification of the contemporary STEC O157:H7 clones. The current models of STEC O157:H7 evolution suggest the sero-conversion of an ancestral *stx2 E. coli* O55 to O157. Subsequent loss of the ability to ferment sorbitol and of β-glucuronidase activity gave rise to the common ancestor of the current circulating clone. The evolutionary models of Leopold *et al.* (2009), Kyle *et al.* (2012) and Yokoyama *et al.* (2012) suggest that the β-glucuronidase-positive last common ancestor may have given rise to lineage II and lineage I/II in a paraphyletic manner with lineage I/II spawning lineage I (with the acquisition of Stx1-containing lambdoid phage seen in clades 1–3 described by Manning *et al.*, 2008). However, strains had previously been identified that confounded these models and indicated that a more complex explanation was needed (Arthur *et al.*, 2013; Mellor *et al.*, 2013).

**Fig. 5.**
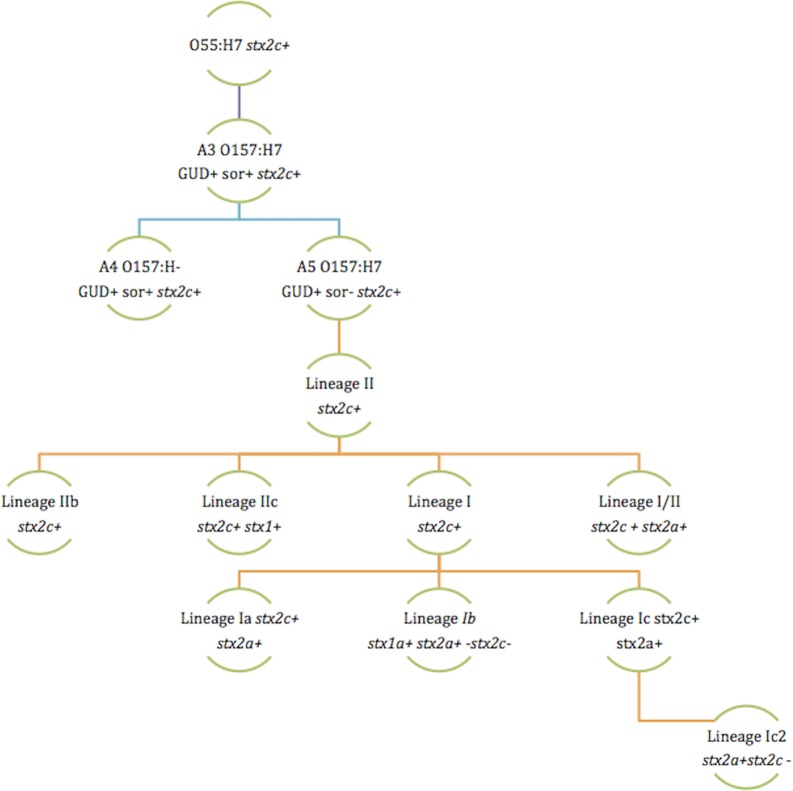
STEC O157:H7 evolutionary model based on a timed phylogeny of over 1000 genomes showing the key evolutionary splits and the associated gain and loss of *stx*-containing prophages. GUD+ represents strains that have the ability to express β-glucuronidase, sor+ represents strains that have the ability to ferment sorbital.

In this study we propose a new evolutionary model based on our phylogenetic analysis (Fig. 5). In this model, we maintain the stepwise series of events from STEC O55 to the β-glucuronidase-positive last common ancestor (A5) that evolved into contemporary lineage II. We show at least three extant lineages of lineage II including the ancestral branch (IIa) as well as a branch that has acquired Stx1ϕ (IIc). A lineage II Stx2cϕ-containing strain independently gave rise to lineage I (approx. 125 years ago) and lineage I/II (approx. 95 years ago). In lineage I/II, a single integration event of a Stx2aϕ into *argW* has been maintained with a subgroup losing Stx2cϕ. Lineage I has a more complex evolutionary history with a Stx2aϕ integrating at least three times (once into *wrbA*, once into *argW*, and once into an unknown site), Stx1ϕ inserting into lineage Ia strains and at least two loss events of the Stx2cϕ. The model presented here shows Stxϕ loss and gain events that have been fixed in the population but we also observed many loss and gain events that appeared to be occurring sporadically within each lineage as well as occupation of SBIs with imported DNA that does not encode Stx. This leads to the conclusion that the loss and gain of phage is likely to be highly dynamic but under high selection for retention in the bovine host. Recombination analysis highlighted the phage regions to be hotspots of DNA exchange, with remarkably little activity outside these regions.

In this analysis we predict the split from the β-glucuronidase-positive last common ancestor (A5) to have occurred approximately 400 years ago with the common ancestor of the current diversity appearing 175 years ago. At this point there was an expansion event with the major lineages formed within 30 or so years. This early diversification of STEC O157:H7 fits with the extant diversity of STEC O157:H7 being globally distributed. Although a large degree of diversity of STEC O157:H7 is seen in the UK, the distribution of this diversity is uneven. We show that several pockets of diversity are seen at much higher frequency then others and that the same pockets of diversity are more frequently observed in both human clinical cases and in the local cattle population. This fits with the model of historical dissemination of diversity and then regional expansion in native cattle with occasional sampling of the wider diversity through imported foodstuff and foreign travel.

Although we have shown the contemporary clone existed over 100 years earlier, STEC O157:H7 only became a recognized pathogen in the 1980s (Riley *et al.*, 1983) after causing outbreaks of severe illness. Whilst STEC O157:H7 causes gastroenteritis in most infections, a significant minority develop more severe symptoms including HUS. Whilst progression to HUS no doubt has many host predictors, a clear association with the presence of *stx2a* subtype has been shown (Persson *et al.*, 2007). In our study, we show that the acquisition of the *stx2a* subtype occurred relatively recently compared with the other *stx* subtypes and is likely to explain the recent emergence of the STEC O157:H7 serotype as a clinically significant pathogen. We also show that *stx2a* is likely to have been acquired by STEC O157:H7 on multiple occasions, highlighting the potential for new, highly virulent clones to emerge. Finally, it appears that once *stx2a* is integrated in a population it tends to be maintained, often at the expense of *stx2c*. Recent research has indicated that the Stx2aϕ is associated not only with more severe human disease but also with higher excretion levels in cattle (Matthews *et al.*, 2013).

Using clinical outcome data on a cohort of nearly 500 STEC O157:H7 cases, we are able to assess the risk of severe disease of each of the extant lineages and sublineages. The presence of *stx2a* is a pre-requisite for the development of HUS with 100 % of HUS cases infected with a strain harbouring this toxin subtype. Multivariable regression analysis with the ancestral IIa clone as the baseline shows IIc has a nearly fourfold increase in risk of severe disease accounted for by in increase in incidence of bloody diarrhoea. This PT8 clone has acquired a Stx1ϕ carrying the same Stx as found in *Shigella dysenteriae* serotype 1. All sublineages of lineage I and I/II that contain *stx2a* have an increased risk of severe disease with the additional presence of *stx2c* appearing to have a protective effect. This presumably reflects regulatory interactions between the prophages. These analyses show the clear importance of determining the Stx complement of an STEC O157 strain when predicting the likely risk of severe disease and, therefore, case management.

This study shows that recent strain replacement has occurred in Great Britain shaping the diversity of STEC O157:H7 observed today. Within lineage II, an importation of a PT8 strain probably from the Mediterranean cattle population of southern Europe and northern Africa occurred within the last 30 years. Similarly, within the last 25 years the emergence and rapid expansion of PT 21/28 in lineage I in Great Britain led to this highly virulent subtype being found ubiquitously in domestic cattle. These recent strain replacement events provide insight into the dynamics of STEC O157:H7 transmission on a national and international scale and suggest that while the overall diversity of this pathogen is globally distributed, regionally endemic strains can be transmitted and eventually become the dominant strain in the local cattle population. Whilst the imported strain may play a role in out-competing domestic strains, agricultural practices such as culling and restocking of animals, as seen during the foot-and-mouth disease and bovine spongiform encephalitis (BSE) epidemics may act as drivers facilitating more rapid strain replacement (Carrique-Mas *et al.*, 2008).

From the current study it appears the relatively high incidence of STEC O157 human infections in the UK results from the emergence and expansion of a lineage I PT21/28 clade in the last 25 years, producing strains containing both Stx2a and Stx2c prophages that are capable of higher excretion levels from cattle (supershedding) and can cause severe disease in humans. Therefore, screening and intervention strategies should be targeting these strain clusters that are the most significant threat to human health. Further work is needed to understand the diversity of host phages that carry Stx and the reasons behind the proliferation of this cluster. While Stx is essential for the severe pathology associated with human STEC disease, the role of the different toxins in governing supershedding is unknown. Moreover, it is evident that other genes on Stx-encoding prophages regulate the expression of bacterial colonization factors, and this will also impact on the success of the cluster (Xu *et al.*, 2012).

## Supplementary Data

42881 Supplementary Data
