# Supplementary material for: Applying phylogenomics to understand the emergence of Shiga-toxin-producing Escherichia coli O157:H7 strains causing severe human disease in the UK
Source: Microb Genom. 2015 Sep 14;1(3):e000029. doi: 10.1099/mgen.0.000029 (PMC5320567; doi:10.1099/mgen.0.000029)
Supplement: Supplementary file 1 — Supplementary Data [file mgen-01-29-s001.pdf]

Phylogenomics of Shiga Toxin producing *Escherichia coli* O157:H7: assessing the risk of severe human disease in light of recent strain replacement in the cattle population in the United Kingdom

Timothy J. Dallman[1*], Philip M. Ashton[1], Lisa Byrne[1], Neil T. Perry[1], Liljana Petrovska[3], Richard Ellis[3], Lesley Allison[5], Mary Hanson[5], Anne Holmes[5], George J. Gunn[7], Margo E. Chase-Topping[6], Mark E. J. Woolhouse[6], Kathie A. Grant[1], David L. Gally[4], John Wain[2*], Claire Jenkins[1].

[1]Public Health England, 61 Colindale Avenue, London, NW9 5EQ
[2]University of East Anglia, Norwich, NR4 7TJ
[3]Animal Laboratories and Plant Health Agency, Woodham Lane, Surrey, KT15 3NB
[4]Division of Infection and Immunity, The Roslin Institute and Royal (Dick) School of Veterinary Studies, University of Edinburgh, Roslin, UK, EH25 9RG.
[5]Scottish *E. coli* O157/VTEC Reference Laboratory, Department of Laboratory Medicine, Royal Infirmary of Edinburgh, 51 Little France Crescent, Edinburgh EH16 4SA.
[6]Centre for Immunity, Infection and Evolution, Kings Buildings, University of Edinburgh, Edinburgh, UK, EH9 3FL.
[7] Future Farming Systems, R&D Division, SRUC, Drummondhill, Stratherrick Rd., Inverness, Scotland, UK, IV2 4JZ

***Corresponding author – tim.dallman@phe.gov.uk**

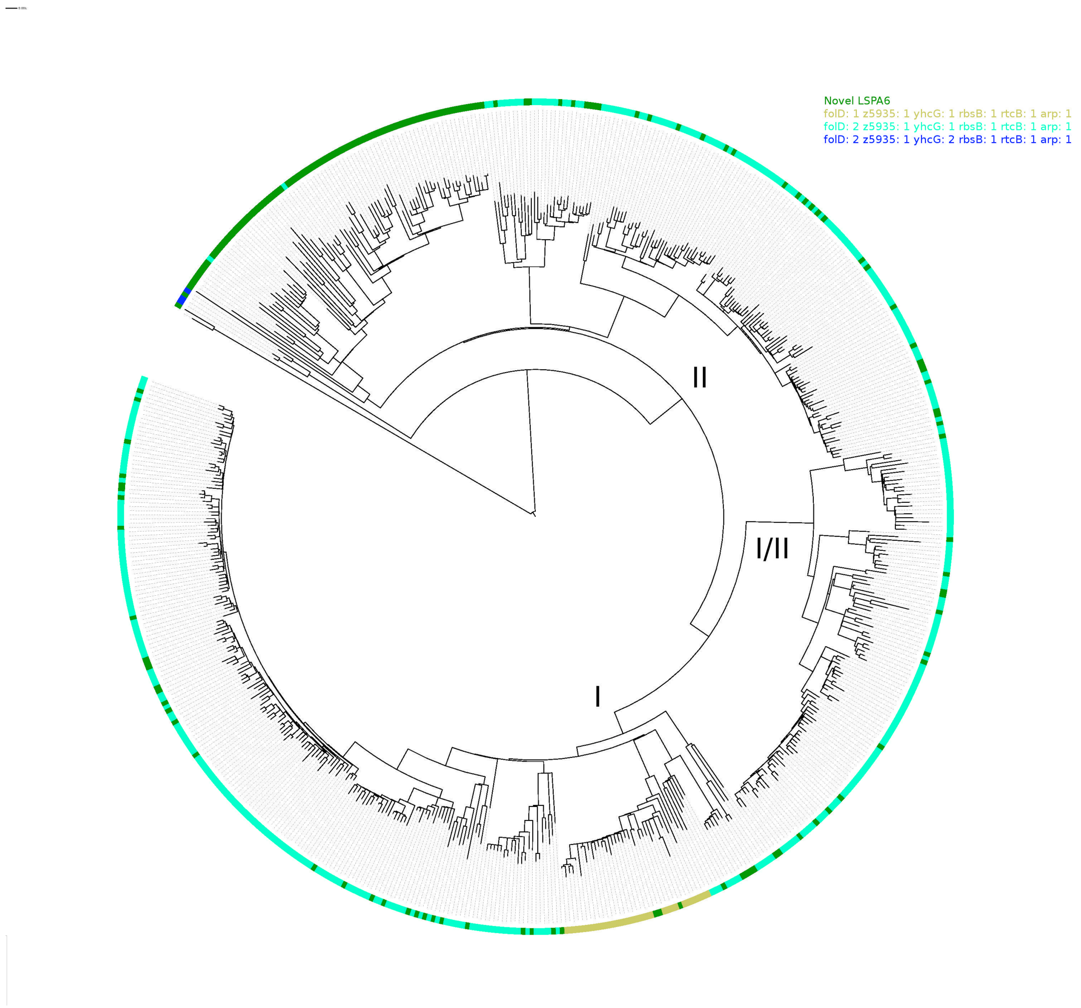

Supplementary Figure 1a.

Maximum likelihood phylogeny of 584 STEC O157:H7 Δ25 SNP representatives depicting the three lineages. The outer circle is coloured by LSPA-6 type.

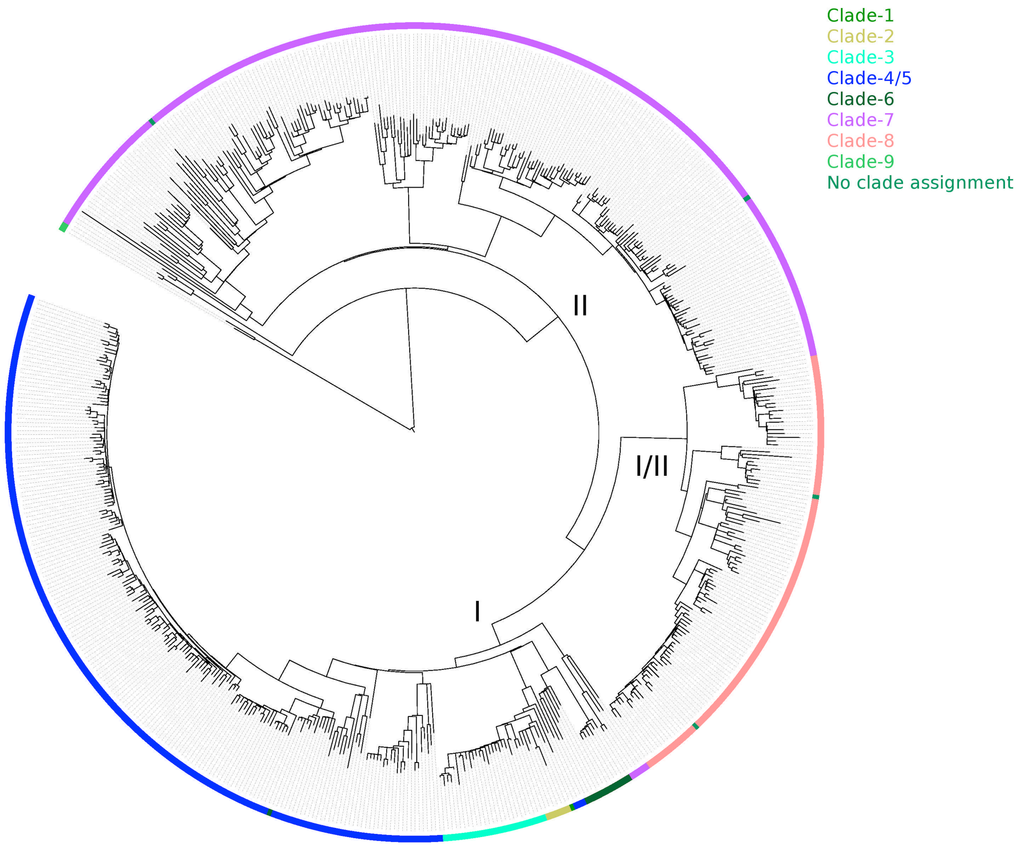

Supplementary Figure 1b.
Maximum likelihood phylogeny of 584 STEC O157:H7 Δ25 SNP representatives depicting the three lineages. The outer circle is coloured by Manning Clade type.

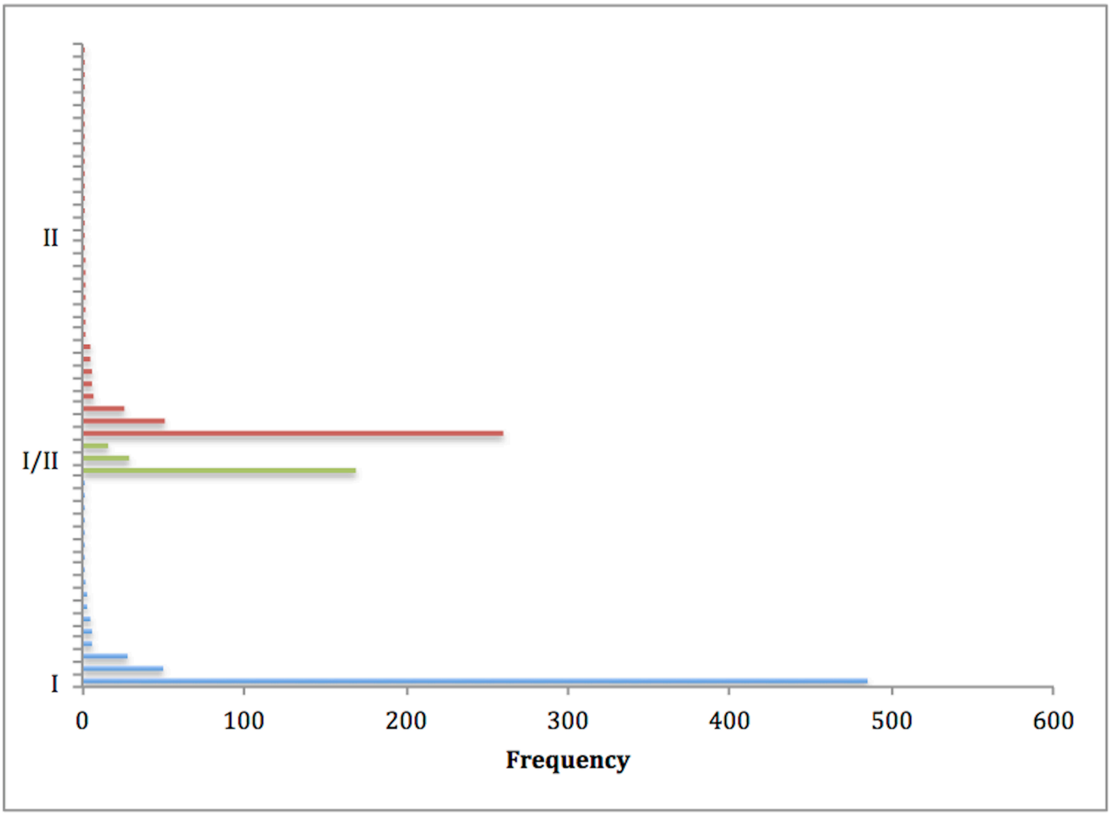

Supplementary Figure 2.
Bar chart showing the number of isolates in each Δ250 SNP cluster.

Lineage	Intra Cluster Recombination	Donor to Lineage I	Donor to Lineage II	Donor to Lineage I/II
**I (828)**	*19.3%*	*56.0%*	*20.3%*	*4.4%*
**I/II (384)**	*29.7%*	*15.1%*	*13.5%*	*41.7%*
**II (1088)**	*18.6%*	*16.5%*	*59.9%*	*5.0%*

Supplementary Table 1.
Table showing the direction of recombination for each of the three lineages of STEC O157:H7. The number in brackets represents the total number of donor segments per lineage.

Lineage II-a	80
**stx 2c**	**65**
yehV-sbcA	53
OTHER	12
**stx negative**	**6**
negative	5
yehV	1
**stx 2a/2c**	**4**
yecE-yehV-sbcA	2
wrbA-yehV-sbcA	1
Z2577-yecE-yehV-sbcA	1
**stx 1a/2c**	**3**
yehV-sbcA-argW	2
yehV-sbcA	1
**stx 2a**	**2**
yecE-yehV-sbcA	2

Lineage II-b	40
**stx 2c**	**20**
yehV-sbcA-argW	15
yehV-sbcA	4
yecE-yehV-sbcA-argW	1
**stx negative**	**8**
negative	8
**stx 2a**	**5**
yehV-sbcA-argW	4
yehV-sbcA	1
**stx 1a/2c**	**4**
sbcA-argW	2
yecE-sbcA-argW	1
yehV-sbcA-argW	1
**stx 2a/2c**	**3**
yehV-sbcA-argW	3

Lineage II-c	250
**stx 1a/2c**	**234**
yehV-sbcA	156
yehV-sbcA-argW	61
OTHER	17
**stx 1a/2a/2c**	**6**
yehV-sbcA	4
yecE-yehV-sbcA	2
**stx 1a**	**5**
yehV	5
**stx 2a/2c**	**3**
yehV-sbcA	3
**stx 2a**	**1**
yehV-sbcA-argW	1
**stx 2c**	**1**
yehV-sbcA	1

Supplementary Table 2:
The proportion of stx sub-type and occupied stx-associated bacteriophage insertion site (SBI) for each sub-lineage of lineage II. Those SBI's that less than 10% of the total were grouped into an 'other' category.

Lineage I/II	167
**stx 2a/2c**	**109**
yehV-sbcA-argW	88
OTHER	21
**stx 2a**	**55**
yehV-argW	44
yehV-sbcA-argW	6
OTHER	5
**stx 2c**	**3**
yecE-yehV-sbcA	2
yehV-argW	1

Supplementary Table 3:
The proportion of stx sub-type and occupied stx-associated bacteriophage insertion site (SBI) for lineage I/II. Those SBI's that less than 10% of the total were grouped into an 'other' category.

Lineage I-a	41
**stx 1a/2a**	**26**
wrbA-yehV	23
OTHER	3
**stx 2a/2c**	**6**
yehV-sbcA	6
**stx 2c**	**5**
yehV-sbcA	4
yecE-yehV-sbcA	1
**stx 2a**	**2**
wrbA-yehV	1
yecE-wrbA	1
**stx 1a**	**1**
yehV	1
**stx negative**	**1**
negative	1

Lineage I-b	30
**stx 2c**	**26**
yecE-yehV-sbcA	15
yecE-wrbA-yehV-sbcA	6
OTHER	5
**stx 2a/2c**	**1**
yecE-yehV-sbcA-argW	1
**stx negative**	**3**
negative	3

Lineage I-c	467
**stx 2a/2c**	**344**
yehV-sbcA-argW	265
OTHER	79
**stx 2a**	**88**
yehV-sbcA-argW	48
yecE-yehV	22
OTHER	18
**stx 2c**	**30**
yehV-sbcA	17
yehV-sbcA-argW	5
OTHER	8
**stx 1a/2c**	**3**
sbcA	1
yehV-sbcA	1
Z2577-yecE-wrbA-yehV-sbcA	1
**stx negative**	**2**
yehV-sbcA-argW	2

Supplementary Table 4:
The proportion of stx sub-type and occupied stx-associated bacteriophage insertion site (SBI) for lineage I. Those SBI's that less than 10% of the total were grouped into an 'other' category.

Supplementary Table 5:
All 1129 genomes analysed in this study summarised in terms of Lineage, SNP cluster, SBI, *stx* type, Manning Clade and LSPA-6 type.
